# Recovery from resistance exercise in older adults: a protocol for a scoping review

**DOI:** 10.1136/bmjsem-2021-001229

**Published:** 2022-01-31

**Authors:** Eleanor Jayne Hayes, Emma Stevenson, Avan Aihie Sayer, Antoneta Granic, Christopher Hurst

**Affiliations:** 1 AGE Research Group, Translational and Clinical Research Institute, Newcastle University, Newcastle upon Tyne, UK; 2 Population Health Sciences Institute, Newcastle University, Newcastle upon Tyne, UK; 3 NIHR Newcastle Biomedical Research Centre, Newcastle upon Tyne Hospitals NHS Foundation Trust and Newcastle University, Newcastle upon Tyne, UK

**Keywords:** review, ageing, exercise, weight lifting, DOMS

## Abstract

**Introduction:**

Resistance exercise has been shown to improve muscle health in older adults and is recommended as a front-line treatment for many health conditions, including sarcopenia and frailty. However, despite considerable research detailing the potential benefits of resistance exercise programmes, little is known about how older adults recover from individual exercise sessions. This scoping review will examine the current evidence surrounding the acute post-exercise effects of resistance exercise and the exercise recovery process in older adults to inform future research and exercise prescription guidelines for older adults.

**Methods and analysis:**

The methodological framework of Arksey and O’Malley (2005) will be applied for this scoping review. A systematic search of five online databases and the hand-searching of reference lists of identified articles will be used to identify relevant papers. Studies that aim to measure exercise-induced muscle damage or exercise recovery following a resistance exercise session in participants aged 65 years and over will be included. Qualitative and quantitative data from relevant studies will be presented in a tabular format. Results will be summarised in narrative format. Key findings will be discussed concerning resistance exercise prescription in older adults.

**Dissemination:**

This review will be used to direct further research surrounding the exercise recovery process from resistance exercise in older adults and will also aid in designing specific exercise prescription guidelines for an older population. Findings will be relevant to researchers, clinicians, health workers and policy-makers and disseminated through publications and presentations.

**Key messages**

**What is already known?**

- Older adults may recover from resistance exercise differently, but there is currently no comprehensive literature review.

**What are the new findings?**

- This novel scoping review will collate research and map the literature surrounding recovery from resistance exercise in older adults.
- The review will bring together literature from both clinical and exercise science disciplines and begin to aid in designing specific exercise prescription guidelines for older populations.

## Introduction

Resistance exercise, also known as strength training or resistance training, has been shown to be effective in improving muscle health in older adults.^1^ After the age of 50, it is thought that muscular strength decreases at a rate of 1.5%–5% per year, inevitably leading to a reduced functional capacity.^2^ There is now compelling evidence that this decline can be attenuated through regular participation in resistance exercise, resulting in improved muscle mass, muscle strength, body composition and metabolic health.^3^ Moreover, these adaptations have been shown to reduce fall risk,^4^ prevent disability and improve quality of life in older adults.^5^ Consequently, resistance exercise is recommended as a front-line treatment for many health conditions, including sarcopenia,^2 6^ a skeletal muscle disorder characterised by progressive loss of muscle strength and mass,^7^ and frailty. However, despite considerable research describing the potential benefits of resistance exercise in older adults, exercise recovery and the process of muscle repair and adaptation following intense exercise are distinctly understudied. Indeed, little is known regarding how older adults recover from individual exercise sessions that may be performed as part of a resistance training programme.

Despite the potential long-term benefits, performing resistance exercise may cause unfavourable short-term side effects as a result of transient fatigue and exercise-induced muscle damage. Exercise-induced muscle damage typically occurs due to unaccustomed or intense exercise. It is characterised by muscle soreness and a loss of skeletal muscle function immediately after and in the days following an exercise bout.^8^ Alongside impacting functional capacity, exercise-induced muscle damage can also reduce exercise capacity and interfere with the sense of force production and limb position^9^ for up to 14 days after exercise.^8^ To directly measure damage to the skeletal muscle, assessment of a muscle biopsy under a microscope is required. However, the procurement of a muscle biopsy is not always feasible, practical or ethical. Therefore, symptoms of muscle damage are often used to quantify the extent of muscle damage indirectly.^10^ These symptoms include loss of muscle strength and power, muscle soreness, increased limb circumference and blood markers of muscle damage such as creatine kinase and myoglobin.^11–13^ Generally, these symptoms are caused both by structural damage within the skeletal muscle occurring during the exercise and by a prolonged inflammatory response in the following days.^8 11^ The duration and magnitude of these symptoms are variable. They depend not only on the training intensity,^14^ the muscles used^15^ or the contraction type.^16^ They may also be heavily influenced by individual characteristics such as training status,^17^ nutrition,^18^ sex^19^ and age.^20^ These symptoms may be particularly impactful in older adults where there is already some age-related decline in functional capacity.

Traditionally, resistance exercise has been performed by athletes to improve physical performance. As such, a large proportion of the literature surrounding exercise recovery and muscle damage is concentrated on younger adults. According to the theory of adaptation,^21 22^ the recovery period is considered highly important when prescribing exercise programmes that aim to produce distinct physiological changes through repeating cycles of muscle damage and repair. Generally, literature in younger adults seeks to understand the muscle damage and recovery cycle to optimise training adaptations for sporting performance, but this may not be as relevant for an older population. Indeed, some literature suggests that ageing results in greater muscle damage and that the time course of the exercise recovery process could be prolonged with age.^23^ These potential differences cast doubt on the applicability of pre-existing research with younger participants. Additionally, advancing age presents a range of alternative considerations when designing exercise programmes. The psychological^24^ and physiological^25^ way in which exercise-induced muscle damage is experienced may present challenges to older adults that are not deemed relevant in younger individuals.

The main aim of resistance exercise for older adults is maintaining muscle mass and strength. Yet, any transient decrements in physical functioning resulting from intense exercise in these individuals could hinder their ability to perform habitual activities such as climbing a flight of stairs or acutely increase their risk of falls.^26 27^ Therefore, while optimising exercise prescription for training adaptations remains essential, we must also consider the effects of resistance exercise on individuals with lower physical functioning and their ability to cope with symptoms of muscle damage in the days following exercise. A greater understanding of this topic is needed to establish how these symptoms may interact with the daily lives of older individuals, the optimal frequency of resistance training considering the time course of exercise recovery, and how they affect continued engagement with resistance exercise. Indeed, optimising training adaptations to maintain muscle strength with advancing age while mediating any adverse effects of exercise-induced muscle damage should be an important aim of all exercise prescriptions for older adults.

This scoping review will map the current evidence surrounding exercise-induced muscle damage and recovery from resistance exercise in older adults. Specifically, this review aims to establish; (a) which population groups have been studied; (b) how the exercise recovery process has been characterised; (c) what acute post-exercise effects of resistance training have been documented in older adults; (d) the time course of exercise recovery in older adults and (e) what variables (if any) have been shown to alter the exercise recovery process in this population.

## Methods and analysis

This review protocol will be reported in accordance with the guidance from the Preferred Reporting Items for Systematic Reviews and Meta-Analyses extension for Scoping Reviews (PRISMA-ScR).^28^ The review will report on the current state of the literature regarding the acute effects of resistance exercise in older adults. It will follow the framework for scoping reviews first outlined by Arksey and O’Malley^29^ and further refined by the Joanna Briggs Institute.^30^ This framework requires the following steps: (a) identifying the research question; (b) identifying relevant studies; (c) study selection; (d) charting of the data and (c) collating, summarising and reporting the results.

### Identifying the research question

The overall aim of this review is to assess the current evidence surrounding exercise-induced muscle damage, and recovery from resistance exercise, in older adults. The research questions have been defined as follows:

1. Which population groups have been included in current research (eg, age, sex, training status) and what are the key characteristics of the resistance exercise undertaken (eg, intensity, volume, contraction type)?
2. How has recovery been characterised? What are the key outcomes (both subjective and objective) that have been measured to quantify exercise-induced muscle damage and the exercise recovery process?
3. What are the acute post-exercise effects of resistance exercise, including physiological markers of exercise-induced muscle damage and ability to complete daily tasks, and what is the time course for these effects?
4. Which variables have been shown to affect the extent of muscle damage and the time course of the exercise recovery process in an older population (eg, individual characteristics, nutrition, other recovery strategies)?

We define the key terms of the research questions as follows:

- *Resistance exercise*: any physical activity that produces skeletal muscle contraction(s) by using an external resistance or weight.
- *Exercise-induced muscle damage:* A temporary disturbance in the function or structure of skeletal muscles as a result of intense or unaccustomed exercise
- *Exercise recovery:* The process of resolving physiological and psychological disturbances that may occur due to performing an intense or unaccustomed exercise. This process begins once exercise has ceased.

For this review, we define the period during which exercise recovery occurs immediately after and up to 7 days following the exercise session. We acknowledge that some symptoms of muscle damage may extend past this time frame after very intense exercise, but this is unlikely to be representative of exercise performed by an older population. This time frame will also allow us to ensure we are identifying studies that measure exercise recovery instead of chronic training adaptations.

### Identifying relevant studies

The following electronic databases will be searched using MeSH terms and free text: MEDLINE, Scopus, Embase, SPORTDiscus and Web of Science. In addition, reference lists of all identified articles will be screened for additional studies.

The search strategy will include terms related to the population of interest (ie, adults, older adults, elderly) in combination with the exercise mode (ie, resistance training, weight training, weight lifting, resistance exercise) and the outcomes of interest (ie, muscle damage, exercise recovery, muscle soreness, muscle function, muscle strength, isometric strength, creatine kinase, inflammation, perceived recovery). The full search strategy is available in online supplementary appendix 1. Due to the nature of scoping reviews, the search will be designed to be as broad as possible to minimise the risk of missing relevant material. Studies will be included to meet the eligibility criteria set out in table 1.

10.1136/bmjsem-2021-001229.supp1 Supplementary data

**Table 1:** Study selection criteria

	Inclusion	Exclusion
**P**opulation	Older adults aged 65 years or over	Articles utilising specific clinical populations (eg, patients with cancer).
**I**ntervention	Performance of a resistance training session	There are no restrictions on the intensity of the resistance training or the muscle groups used during this exercise.
**C**omparison	No comparator group is necessary	Nil exclusion criteria.
**O**utcome	All direct and indirect measures of acute muscle damage and exercise recovery, including but not limited to; muscle strength, physical functioning, muscle soreness, muscle power, perceived recovery, creatine kinase, inflammation, myoglobin, range of motion and limb circumference. Ultrastructural muscle damage will also be considered as an outcome of interest.	Nil exclusion criteria.
Publication Type	Published primary research studies, including both qualitative and quantitative research. Abstracts of unpublished studies for which authors can be contacted and provide sufficient information to enable accurate analysis.	Literature reviews. Systematic reviews. Meta-analyses. Trial protocols. Book chapters. Text. Conference abstracts. Opinion papers. Letters. They are not published in English.

All primary research articles that meet the criteria will be included in the review. Studies that contain older adults of both sexes from all races/ethnicities, settings and geographical areas will be included. We will not exclude studies that contain a younger age group in addition to older adults. Although we will not require studies to exclusively include healthy, disease-free older adults, studies that have been conducted within specific clinical populations will be excluded. We will also exclude trials where there is no ‘exercise only’ condition (ie, where all conditions have received a recovery intervention) and where there is, therefore, no data on the participants usual response to resistance training.

Different outcomes than those stated in table 1 may be used to measure exercise-induced muscle damage or exercise recovery. Any other parameter not mentioned in table 1 that two reviewers agree to be assessing exercise-induced muscle damage or exercise recovery will be included. We will consider any method used to measure these outcomes in the included studies (eg, for physical functioning, we will accept both the chair stand test and the timed up-and-go test).

### Study selection

All eligible articles will be uploaded to Zotero 5.0, where duplicate articles will be removed. The authors will conduct the initial screening of the articles. To ensure the suitability of the selected studies for the research objectives, two reviewers (EJH and CH) will screen by title and abstract. Any excluded studies will be reviewed by a third reviewer (AG). If the eligibility of a study is not clear from the abstract, a full-text article will be obtained. Full-text screening of the subsequently selected articles will be conducted by two reviewers independently (EJH and CH). Every effort will be made to obtain full-text articles of the selected articles, including web searching, contacting the necessary authors and consultation of a university librarian. Discrepancies between reviewers will initially be sought to be rectified by discussion. In the case of no resolution, a third reviewer (AG) will be asked to determine a consensus.

### Charting of the data

Data will be extracted from all eligible studies using a standardised form to chart the data developed by three reviewers (EJH, CH and AG). As is the nature of a scoping review, this form may need continually reviewing by the reviewers throughout the data extraction process to ensure all relevant information is gathered to address the aim of the scoping review successfully. This form aims to gather all of the relevant information surrounding the exercise recovery process in older adults. Data will be charted by one author (EJH) and checked by a second author (CH). Any disagreements will be resolved first by discussion between the two reviewers or further adjudicated by the third (AG) if a unanimous decision is not made.

Information of interest will include the following:

- Study characteristics: year of publication, journal, aims and objectives of the study, study design, sample size, country of origin, study setting
- Participant characteristics: population sampled, age (eg, mean with SD and range), sex (eg, percentage of male/female participants), training status (eg, untrained or resistance-trained).
- Resistance exercise intervention protocol (eg, exercises performed, muscle groups used, training intensity, training volume, contraction type and other relevant information).
- Outcome results (eg, finding relevant to exercise recovery or exercise-induced muscle damage)
- The time frame of outcome measures (eg, at what time-points were data collected in relation to the resistance exercise protocol)
- Presence of any comparison groups (eg, young adults or a nutritional intervention).
- Key relevant findings and conclusions

### Collation, summarisation, and reporting of the results

A PRISMA flow diagram will be used to report the final numbers of relevant articles included in the review. Both quantitative and qualitative data from relevant studies will be presented in a tabular format, with further narrative descriptions if necessary. The study findings will be synthesised in a narrative format based on the research questions and any themes identified during data extraction. The implications of these findings and identified gaps in the literature will also be discussed in narrative form.

Our key findings, alongside other relevant exercise science literature, will be discussed in relation to the prescription of resistance exercise in older adults. The review will also highlight the key areas where additional or better-quality evidence is needed before specific recommendations regarding exercise recovery in older adults can be made.

### Patient and public involvement

There will be no patient or public involvement in our scoping review.

## Ethics and dissemination

The completed scoping review will be submitted for publication in a peer-reviewed journal and contribute to a PhD thesis. Any findings from this review will also be disseminated at appropriate conferences. Findings from this review can be used to direct further research surrounding the exercise recovery process from resistance training in older adults. Still, they may also begin to inform specific exercise prescription guidelines for an older population. Only secondary data will be used for this review, and therefore ethical approval is not required.

## Discussion

The short-term post-exercise effects of resistance exercise are poorly understood in older adults. This scoping review will assess the current knowledge of exercise-induced muscle damage and recovery from resistance exercise in older people. Given the unique challenges that can be presented by older age, such as reduced functional capacity, ability to perform day-to-day activities and increased falls risk, this review will aim to apply its findings in a context that is relevant for older adults. It will also highlight gaps in the literature investigating how the exercise recovery process may be altered by training status or nutrition. Overall, this review will aim to bring together literature from both clinical and exercise science disciplines and begin to inform practical recommendations for implementing resistance exercise in an older population. This review can increase awareness of the varying needs of older adults in a post-exercise setting and will also provide a catalyst for interdisciplinary conversation and research aiming to establish better guidelines for exercising older adults.

A potential limitation of this scoping review is that no firm conclusions may be drawn regarding best practices for prescribing resistance training in older adults. Indeed, due to a lack of rigorous quality appraisal of studies, this review is intended simply to map the extent of the literature and begin to discuss possible implications for exercise prescription.

## Data Availability

Data sharing not applicable as no datasets generated and/or analysed for this study. Not applicable.
