# Supplementary material for: Recovery from resistance exercise in older adults: a protocol for a scoping review
Source: BMJ Open Sport Exerc Med. 2022 Jan 31;8(1):e001229. doi: 10.1136/bmjsem-2021-001229 (PMC8804680; doi:10.1136/bmjsem-2021-001229)
Supplement: Supplementary data [file bmjsem-2021-001229supp001.pdf]

Online Supplementary Appendix 1.
Search Strategy

We will systematically search the following electronic databases for studies: MEDLINE, Scopus, Embase, SPORTDiscus and Web of Science. In addition, reference lists of all identified articles will be screened for additional studies.

The search strategy will include terms related to the population of interest (i.e. adults, older adults, elderly) in combination with the exercise mode (i.e. resistance training, weight training, weight-lifting, resistance exercise) and the outcomes of interest (i.e. muscle damage, exercise recovery, muscle soreness, muscle function, muscle strength, isometric strength, creatine kinase, inflammation, perceived recovery).

Hayes EJ, *et al. BMJ Open Sp Ex Med* 2022; 8:e001229. doi: 10.1136/bmjsem-2021-001229

MedLine (Ovid MEDLINE(R) and In-Process & Other Non-Indexed Citations 1946 to Present)

▲ Searches

1	exp Resistance Training/ or exp Weight Lifting/
2	("strength training" or "resistance exercise" or "eccentric exercise" or "lengthening contractions").mp. [mp=title, abstract, original title, name of substance word, subject heading word, floating sub-heading word, keyword heading word, organism supplementary concept word, protocol supplementary concept word, rare disease supplementary concept word, unique identifier, synonyms]
3	1 or 2
4	exp "Aged, 80 and over"/ or exp Aged/
5	("older adults" or "elderly" or "masters athletes" or "veteran" or "post-menopausal" or "older" or "old").mp. [mp=title, abstract, original title, name of substance word, subject heading word, floating sub-heading word, keyword heading word, organism supplementary concept word, protocol supplementary concept word, rare disease supplementary concept word, unique identifier, synonyms]
6	4 or 5
7	3 and 6
8	("muscle dysfunction" or "exercise induced muscle damage" or "muscle damage" or "muscle soreness" or "exercise recovery" or "fatigue" or "recovery" or "creatine kinase" or "myoglobin" or "responses" or "Doms").mp. [mp=title, abstract, original title, name of substance word, subject heading word, floating sub-heading word, keyword heading word, organism supplementary concept word, protocol supplementary concept word, rare disease supplementary concept word, unique identifier, synonyms]
9	7 and 8

Hayes EJ, *et al. BMJ Open Sp Ex Med* 2022; 8:e001229. doi: 10.1136/bmjsem-2021-001229

EMBASE (Embase 1974 to Present)

Searches	Results
1	exp Resistance Training/ or exp Weight Lifting/
2	("strength training" or "resistance exercise" or "eccentric exercise" or "lengthening contractions").mp. [mp=title, abstract, heading word, drug trade name, original title, device manufacturer, drug manufacturer, device trade name, keyword, floating subheading word, candidate term word]
3	1 or 2
4	exp "Aged, 80 and over"/ or exp Aged/
5	("older adults" or "elderly" or "masters athletes" or "veteran" or "post-menopausal" or "older" or "old").mp. [mp=title, abstract, heading word, drug trade name, original title, device manufacturer, drug manufacturer, device trade name, keyword, floating subheading word, candidate term word]
6	4 or 5
7	3 and 6
8	("muscle dysfunction" or "exercise induced muscle damage" or "muscle damage" or "muscle soreness" or "exercise recovery" or "fatigue" or "recovery" or "creatine kinase" or "myoglobin" or "responses" or "Doms").mp. [mp=title, abstract, heading word, drug trade name, original title, device manufacturer, drug manufacturer, device trade name, keyword, floating subheading word, candidate term word]
9	7 and 8

Scopus

ID	Query
result #4	(TITLE-ABS-KEY ("strength training" OR "resistance exercise" OR "eccentric exercise" OR "lengthening contractions" OR "weight training" OR "resistance training" OR "weight lifting")) AND (TITLE-ABS-KEY ("muscle dysfunction" OR "muscle damage" OR "muscle soreness" OR "exercise recovery" OR "fatigue" OR "recovery" OR "creatine kinase" OR "myoglobin" OR "Doms" OR "responses" OR "exercise induced muscle damage")) AND (TITLE-ABS-KEY ("older adults" OR "elderly" OR "veterans" OR "masters athletes" OR "post-menopausal" OR "older" OR "old" OR "aged"))
result #3	TITLE-ABS-KEY ("older adults" OR "elderly" OR "veterans" OR "masters athletes" OR "post-menopausal" OR "older" OR "old" OR "aged")
result #2	TITLE-ABS-KEY ("muscle dysfunction" OR "muscle damage" OR "muscle soreness" OR "exercise recovery" OR "fatigue" OR "recovery" OR "creatine kinase" OR "myoglobin" OR "Doms" OR "responses" OR "exercise induced muscle damage")
result #1	TITLE-ABS-KEY ("strength training" OR "resistance exercise" OR "eccentric exercise" OR "lengthening contractions" OR "weight training" OR "resistance training" OR "weight lifting")

SportDiscus

Search ID#	Search Terms	Search Options	Actions
	S4	S1 AND S2 AND S3	**Expanders** - Apply equivalent subjects<br>**Search modes** - Boolean/Phrase
	S3	("muscle dysfunction" or "exercise induced muscle damage" or "muscle damage" or "muscle soreness" or "exercise recovery" or "fatigue" or "recovery" or "creatine kinase" or "myoglobin" or "Doms" or "responses")	**Limiters** - Language: English<br>**Expanders** - Apply equivalent subjects<br>**Search modes** - Boolean/Phrase
	S2	("older adults" or "elderly" or "masters athletes" or "veteran" or "post-menopausal" or "older" or "old" or "aged")	**Limiters** - Language: English<br>**Expanders** - Apply equivalent subjects<br>**Search modes** - Boolean/Phrase
	S1	("strength training" or "resistance exercise" or "eccentric exercise" or "lengthening contractions" or "resistance training" or "weight training" or "weight lifting" or "isometric exercise")	**Limiters** - Language: English<br>**Expanders** - Apply equivalent subjects<br>**Search modes** - Boolean/Phrase

Web of Science

Set	Save History / Create AlertOpen Saved History
# 4	#3 AND #2 AND #1<br>*Indexes=SCI-EXPANDED, SSCI, A&HCI, CPCI-S, CPCI-SSH, ESCI Timespan=All years*
# 3	**TOPIC:** ("muscle dysfunction" or "exercise induced muscle damage" or "muscle damage" or "muscle soreness" or "exercise recovery" or "fatigue" or "recovery" or "creatine kinase" or "myoglobin" or "Doms" or "responses")<br>*Indexes=SCI-EXPANDED, SSCI, A&HCI, CPCI-S, CPCI-SSH, ESCI Timespan=All years*
# 2	**TOPIC:** ("older adults" or "elderly" or "masters athletes" or "veteran" or "post-menopausal" or "older" or "old" or "aged")<br>*Indexes=SCI-EXPANDED, SSCI, A&HCI, CPCI-S, CPCI-SSH, ESCI Timespan=All years*
# 1	**TOPIC:** ("strength training" or "resistance exercise" or "eccentric exercise" or "lengthening contractions" or "resistance training" or "weight training" or "weight lifting" or "isometric exercise")<br>*Indexes=SCI-EXPANDED, SSCI, A&HCI, CPCI-S, CPCI-SSH, ESCI Timespan=All years*
